# Linearity analysis and comparison study on the epoc^®^ point-of-care blood analysis system in cardiopulmonary bypass patients

**DOI:** 10.1016/j.dib.2016.01.040

**Published:** 2016-01-30

**Authors:** Jianing Chen, Monique Gorman, Bill O’Reilly, Yu Chen

**Affiliations:** a Department of Laboratory Medicine, Dr. Everett Chalmers Regional Hospital, Horizon Health Network, Fredericton, NB, Canada; b Faculty of Medicine, University College Cork, Cork, Ireland; c Department of Laboratory Medicine, Saint John Regional Hospital, Horizon Health Network, Saint John, NB, Canada; d Division of Cardiac Perfusion, Saint John Regional Hospital, Horizon Health Network, Saint John, NB, Canada; e Department of Pathology, Dalhousie University, Halifax, NS, Canada

## Abstract

The epoc^®^ blood analysis system (Epocal Inc., Ottawa, Ontario, Canada) is a newly developed in vitro diagnostic hand-held analyzer for testing whole blood samples at point-of-care, which provides blood gas, electrolytes, ionized calcium, glucose, lactate, and hematocrit/calculated hemoglobin rapidly. The analytical performance of the epoc^®^ system was evaluated in a tertiary hospital, see related research article “Analytical evaluation of the epoc^®^ point-of-care blood analysis system in cardiopulmonary bypass patients” [1]. Data presented are the linearity analysis for 9 parameters and the comparison study in 40 cardiopulmonary bypass patients on 3 epoc^®^ meters, Instrumentation Laboratory GEM4000, Abbott iSTAT, Nova CCX, and Roche Accu-Chek Inform II and Performa glucose meters.

**Specifications table**

**Table:** 

Subject area	Chemistry, Biology
More specific subject area	Point-of-care testing
Type of data	Figure
How data was acquired	The epoc^®^ blood analysis system (Epocal Inc., Ottawa, Ontario, Canada)
Data format	Analyze data
Experimental factors	Patients under cardiopulmonary bypass were all heparinized as routine
Experimental features	Linearity was evaluated using 5 levels of Eurotrol epoc Calibration Verification Fluids and 5 levels of Eurotrol epoc Hematocrit Verification Fluids (Eurotrol B.V., Keplerlaan, The Netherlands) on 3 epoc^®^ blood analysis systems. Linearity materials were analyzed in triplicate on each system. Remnant specimens from cardiopulmonary bypass patients collected in plain 3 mL syringe for routine clinical analysis on GEM4000 in the cardiovascular operating room were used. After being analyzed on GEM4000 and all 3 epoc meters, samples were analyzed in Abbott iSTAT, Nova CCX analyzer, Roche Accu-Chek Inform II and Performa glucose meters side by side, with all measurements performed within 5 min.
Data source location	Saint John, New Brunswick, Canada
Data accessibility	Data are within this article

**Value of the data**

• Detailed analytical linearity analysis for 9 parameters on the epoc^®^ meters was presented.
• Comparison study was conducted on 40 cardiopulmonary bypass patients.
• The data helps medical laboratories and point-of-care testing users to make an informed decision on blood gas analyzer selection.

## 1 Data

**Fig. 1:**
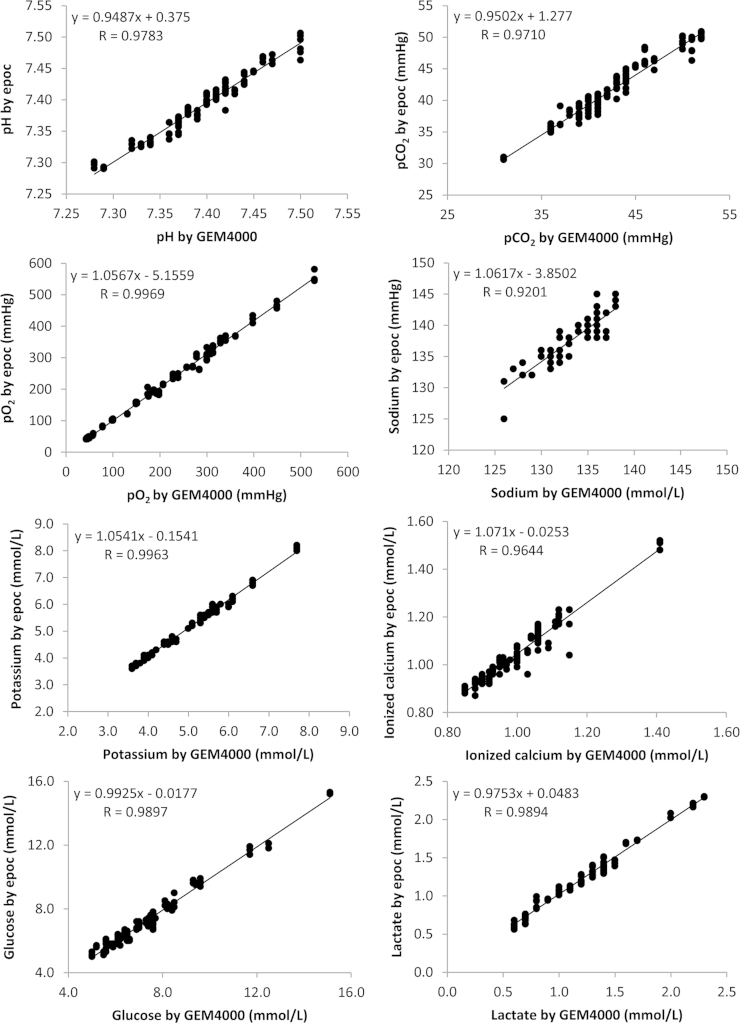
The comparison study of the epoc^®^ point-of-care blood analysis system with the GEM4000 in cardiopulmonary bypass patients.

**Fig. 2:**
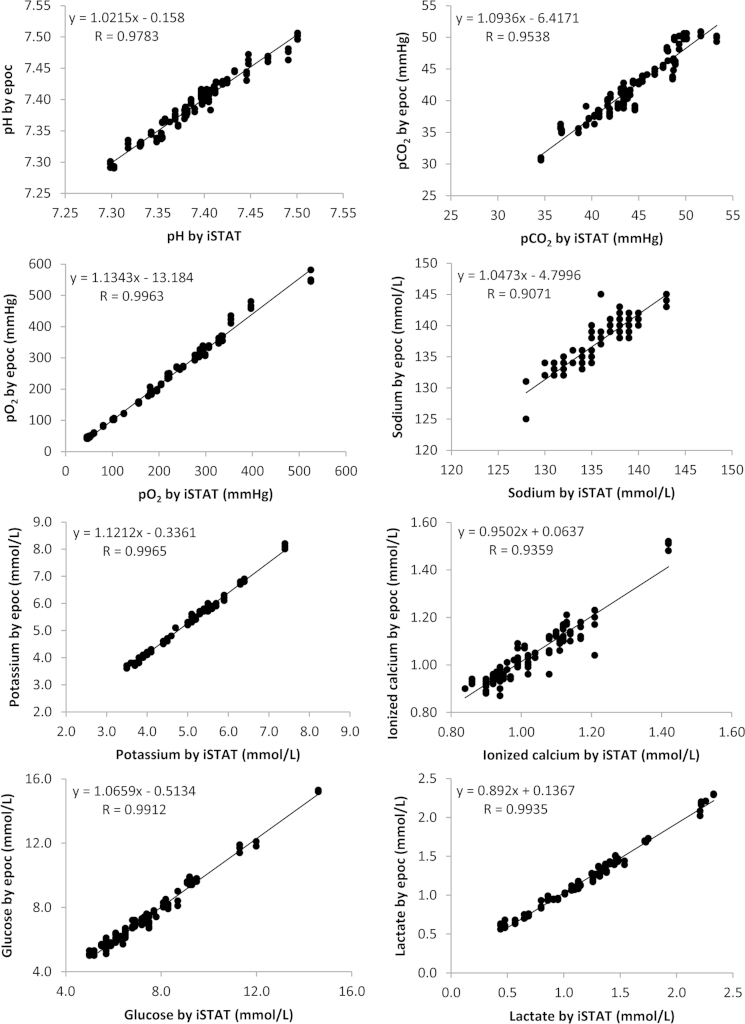
The comparison study of the epoc^®^ point-of-care blood analysis system with the iSTAT in cardiopulmonary bypass patients.

**Fig. 3:**
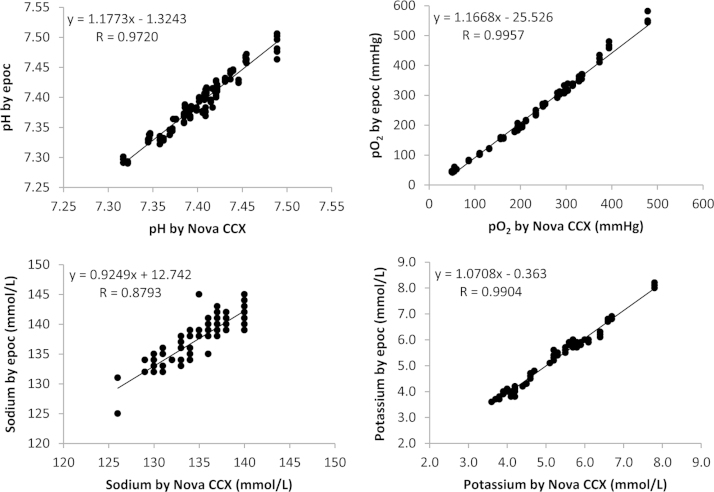
The comparison study of the epoc^®^ point-of-care blood analysis system with the Nova CCX in cardiopulmonary bypass patients.

**Fig. 4:**
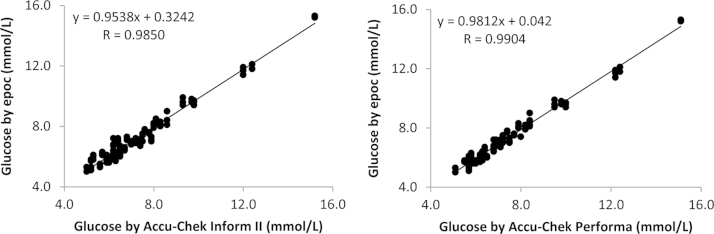
The comparison study of the epoc^®^ point-of-care blood analysis system with the Roche glucose meters in cardiopulmonary bypass patients.

The data contains information on the analytical linearity performances for 9 parameters on 3 epoc^®^ meters (Supplementary Fig. 1). It also contains information on the cardiopulmonary bypass patient sample comparison study for analytical accuracy performance for 8 parameters on 3 epoc^®^ meters (Fig. 1, Fig. 2, Fig. 3, Fig. 4).

## 2 Experimental design, materials and methods

### 2.1 The epoc^®^ blood analysis system

The epoc^®^ blood analysis system (Epocal Inc., Ottawa, Ontario, Canada) is a newly developed hand-held analyzer for testing whole blood samples at point-of-care, which provides blood gas, electrolytes, ionized calcium, glucose, lactate, and hematocrit/calculated hemoglobin in 30 seconds. This system contains a test card, a wireless card reader, and a host mobile computer. pH, pCO_2_, sodium, potassium, and ionized calcium are measure potentiometrically; pO_2_, glucose, and lactate are measured amperometrically, whereas hematocrit is determined conductometrically [2]. Hemoglobin is calculated from the measured hematocrit using the formula: Hemoglobin (g/L)=Hematocrit (decimal fraction)×340 [3], [4].

### 2.2 Linearity study

The epoc^®^ point-of-care blood analysis system was evaluated using several Clinical and Laboratory Standards Institute (CLSI) evaluation protocols for testing the linearity (EP6) [5]. Five levels of Eurotrol epoc Calibration Verification Fluids (Eurotrol B.V., Keplerlaan, The Netherlands, lot#183-B407), and 5 levels of Eurotrol epoc Hematocrit Verification Fluids (Eurotrol B.V., Keplerlaan, The Netherlands, lot#190-B404) were measured on all three epoc^®^ blood analysis systems. These linearity materials were analyzed in triplicate on each system respectively.

### 2.3 Comparison study

The epoc^®^ point-of-care blood analysis system was evaluated using several Clinical and Laboratory Standards Institute (CLSI) evaluation protocols for testing the accuracy (EP15) [6] and bias (EP9) [7]. Remnant specimens from 40 heparinized CPB patients collected in plain 3 mL syringe (Becton Dickinson, Franklin Lakes, New Jersey) for routine clinical analysis on GEM4000 (Instrumentation Laboratory, Bedford, MA, USA) in the cardiovascular operating room of the Saint John Regional Hospital, Horizon Health Network, were used for this study. Samples collected were of arterial, mixed venous, and venous types. After being analyzed on GEM4000 and all 3 epoc meters (therefore total epoc^®^ measurements were up to 118), samples were analyzed in Abbott iSTAT (Abbott Point of Care, Princeton, NJ, USA), Nova CCX analyzer (Nova Biomedical Corporation, Waltham, MA, USA), Accu-Chek Inform II and Performa glucose meters (Roche Diagnostics, Basel, Switzerland) side by side, with all measurements performed within 5 min. All testing devices were run according to manufacturers’ instructions by a medical laboratory technologist. These arrangements attempted to eliminate pre-analytical errors associated with blood analysis, such as different sample collection containers and sensitive specimen stability [8], [9].

### 2.4 Statistical method

Statistical analysis was carried out using Microsoft Excel. The best fit line by linear regression was used to evaluate assay linearity (Supplementary Fig. 1). Regression analysis was used to evaluate method comparisons (Fig. 1, Fig. 2, Fig. 3, Fig. 4). Bland–Altman analysis was constructed to assess systematic bias between methods (see Ref. [1]). Comparison studies on hemoglobin measurements see Fig. 1 in Ref. [1].
