# Supplementary figures and images for: Linearity analysis and comparison study on the epoc® point-of-care blood analysis system in cardiopulmonary bypass patients
Source: Data Brief. 2016 Jan 30;6:847–52. doi: 10.1016/j.dib.2016.01.040 (PMC4749944; doi:10.1016/j.dib.2016.01.040)

pH

Meter 1

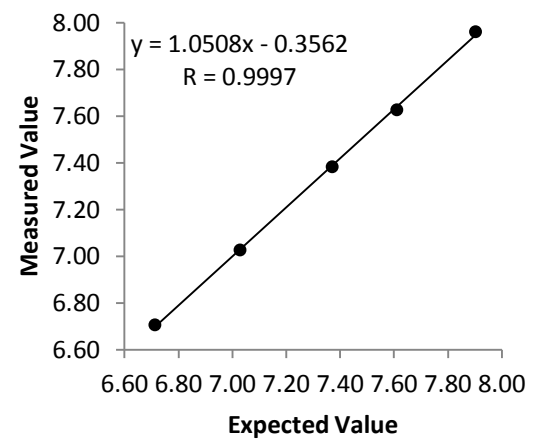

Meter 2

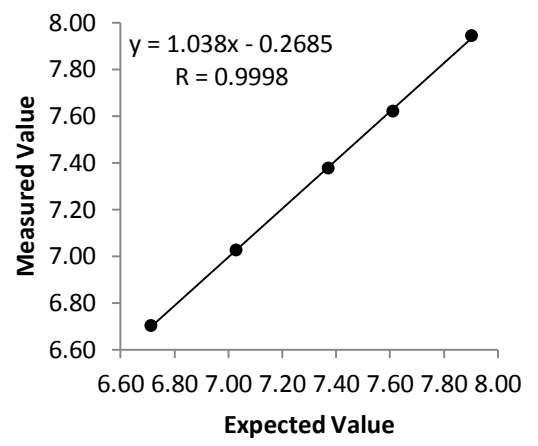

Meter 3

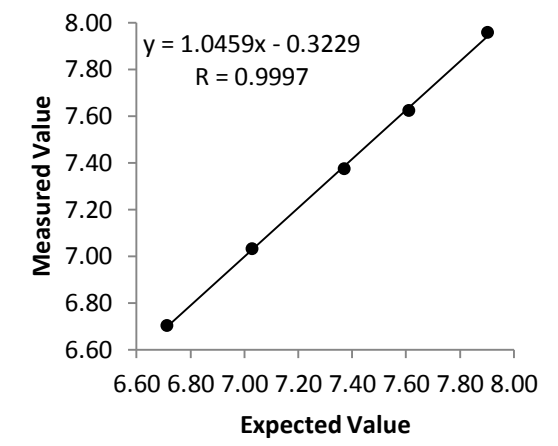

pCO<sub>2</sub>

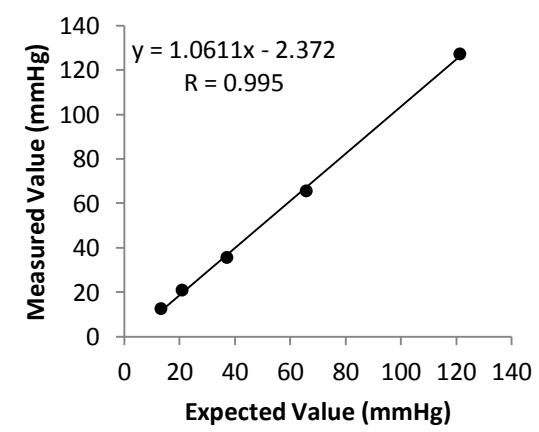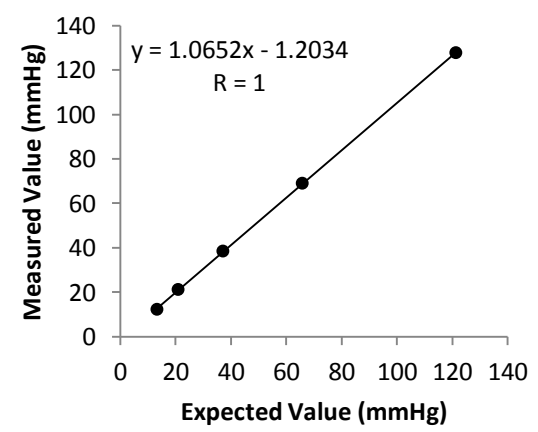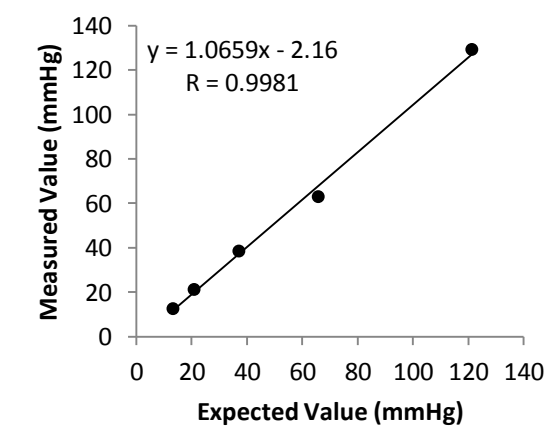

Supplement: Supplementary file 1 — Supplementary material [file mmc1.pdf]

pO<sub>2</sub>

Meter 1

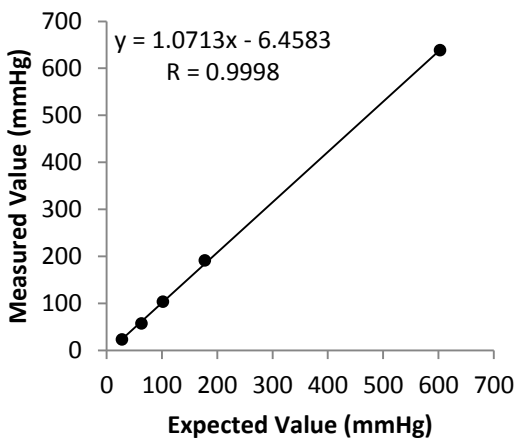

Meter 2

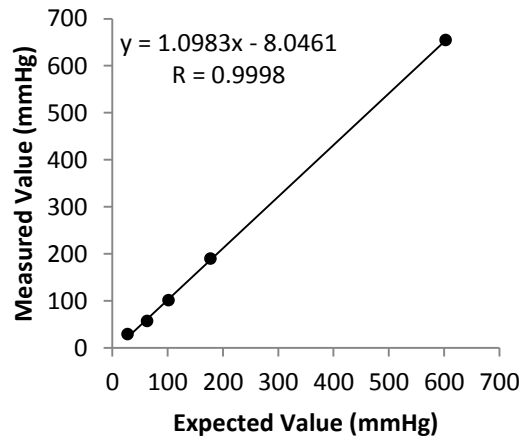

Meter 3

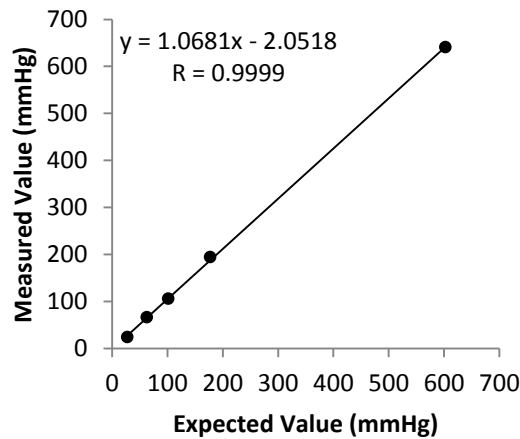

Na<sup>+</sup>

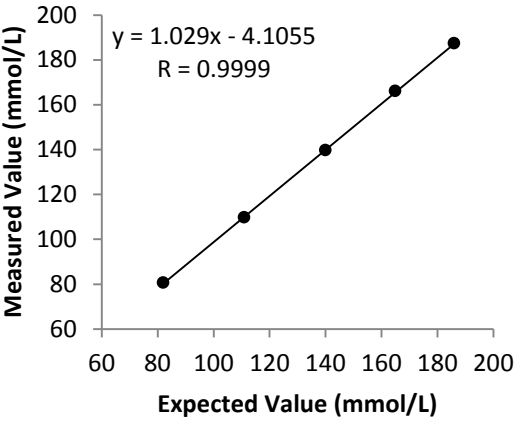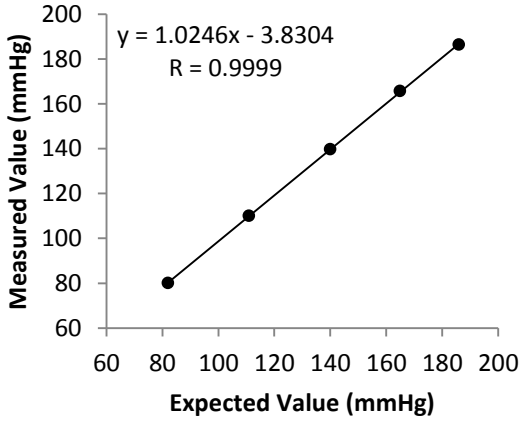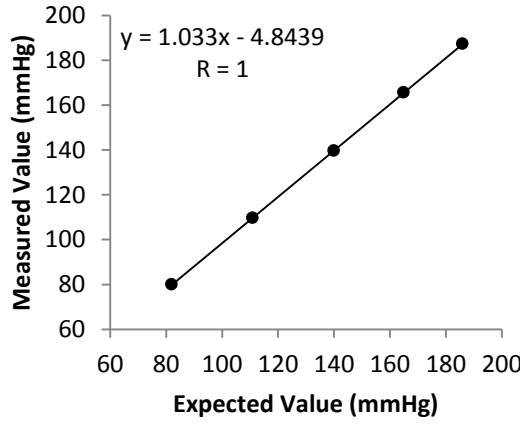

Supplement: Supplementary file 2 — Supplementary material [file mmc2.pdf]

K<sup>+</sup>

Meter 1

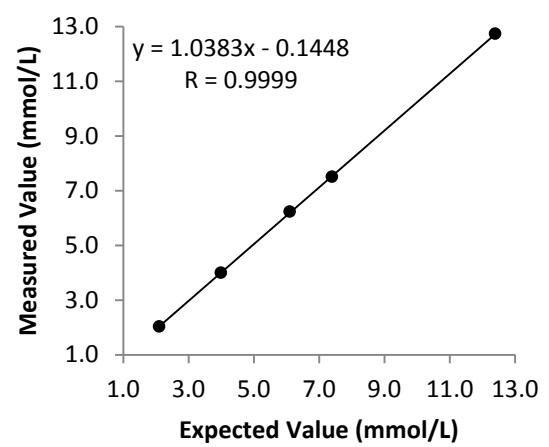

Meter 2

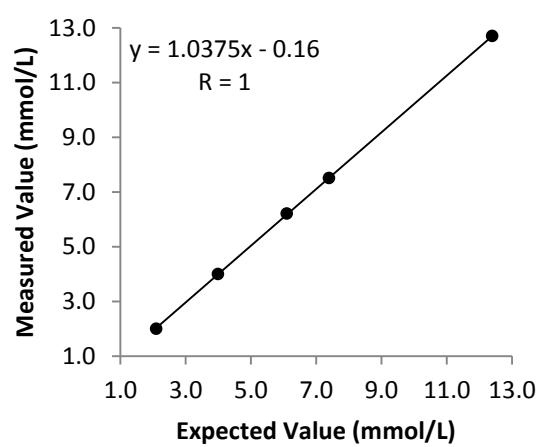

Meter 3

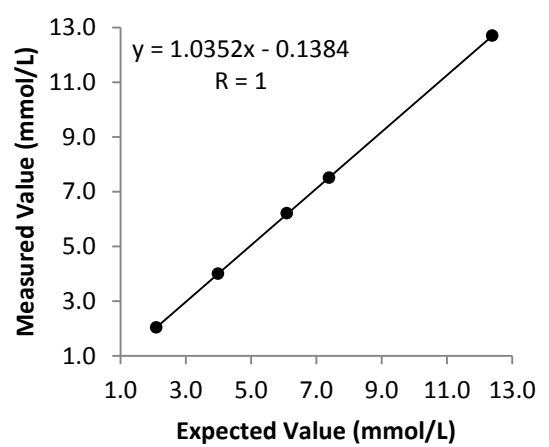

iCa<sup>2+</sup>

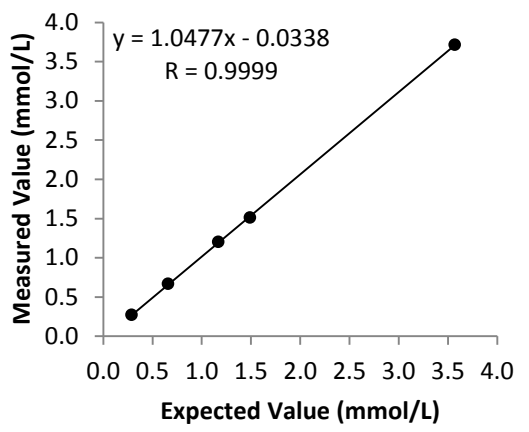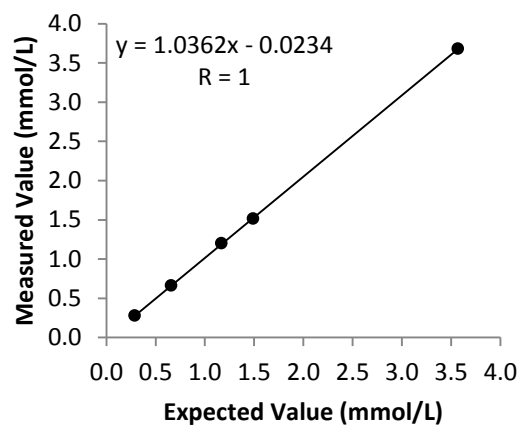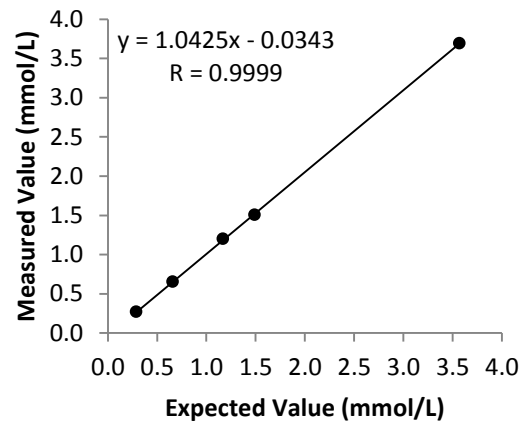

Supplement: Supplementary file 3 — Supplementary material [file mmc3.pdf]

Glucose

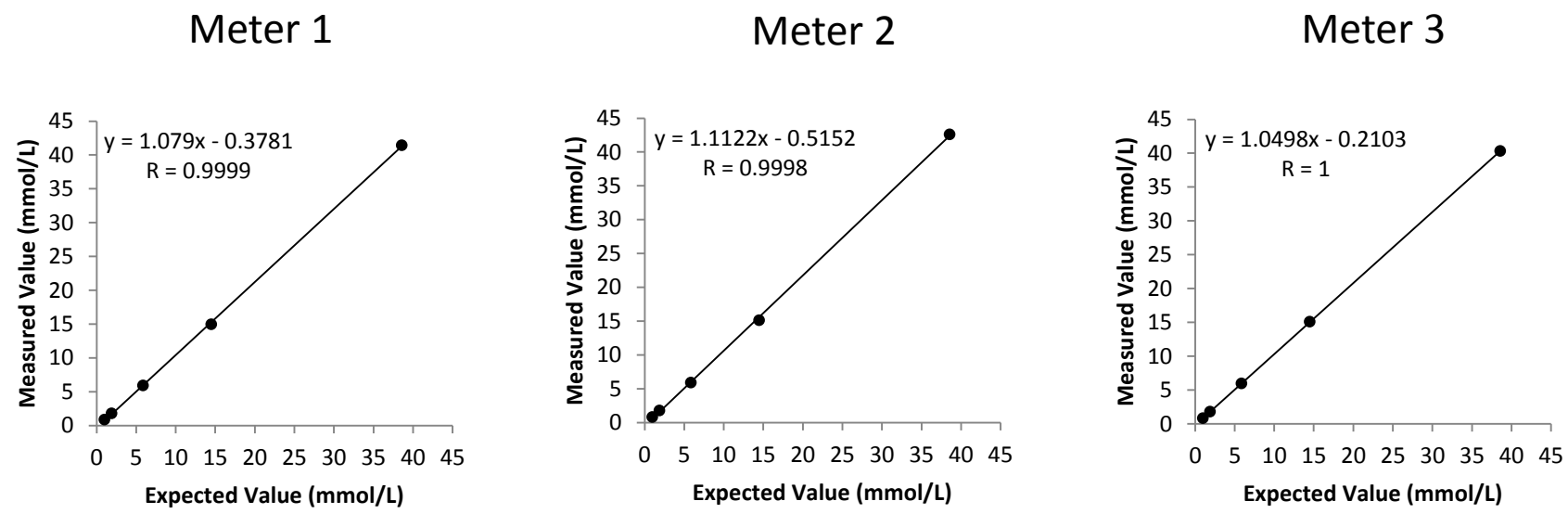

Lactate

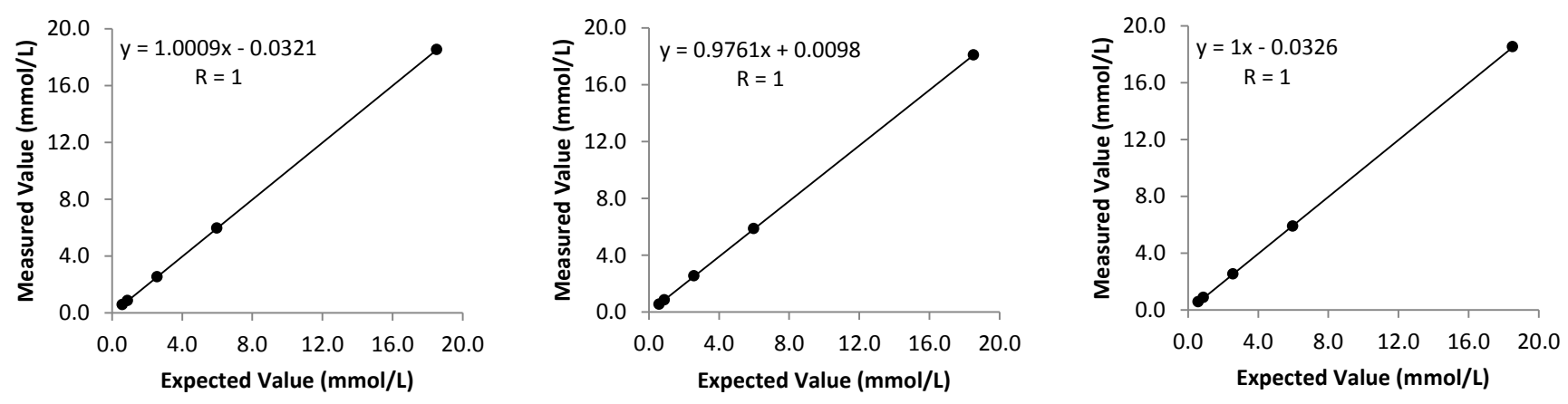

DIB\_Linearity\_Fig. 1\_4

Supplement: Supplementary file 4 — Supplementary material [file mmc4.pdf]

Hct

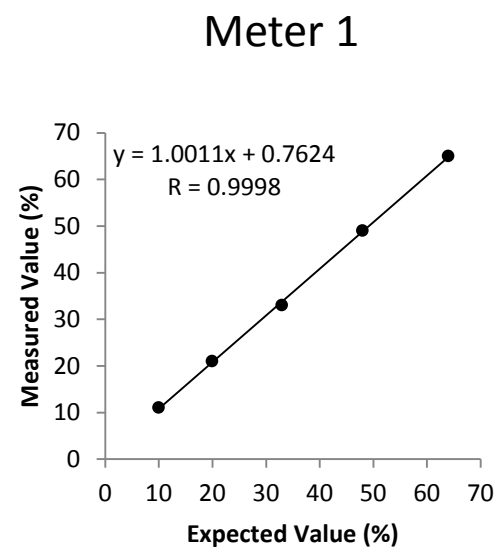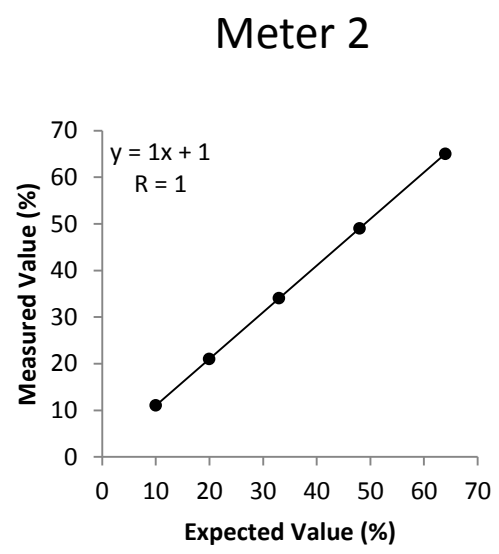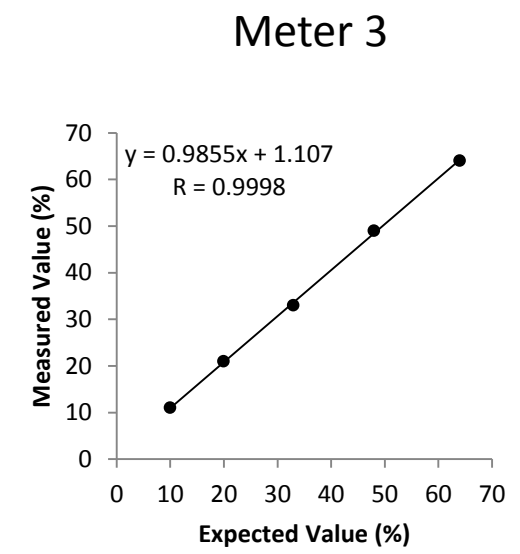

Supplement: Supplementary file 5 — Supplementary material [file mmc5.pdf]
