# Supplementary material for: Linearity analysis and comparison study on the epoc® point-of-care blood analysis system in cardiopulmonary bypass patients
Source: Data Brief. 2016 Jan 30;6:847–52. doi: 10.1016/j.dib.2016.01.040 (PMC4749944; doi:10.1016/j.dib.2016.01.040)
Supplement: Supplementary file 6 — Supplementary material [file mmc6.pdf]

RÉSEAU DE SANTÉ
Horizon
HEALTH NETWORK

January 6, 2015

Anne Ruimy, Ph.D.
Co-EiC
Data in Brief

Dear Dr. Ruimy,

We wish to confirm that there are no known conflicts of interest associated with this publication and there has been no significant financial support for this work that could have influenced its outcome.

Thank you again very much for your consideration of our manuscript for publication.

Sincerely yours,

Yu Chen, MD, PhD, FACB, FCACB, DABCC (Clin Chem, Toxicology)
Medical Biochemist and Division Head, Clinical Biochemistry, Horizon Health Network
Fredericton and Upper River Valley Areas
Email: Yu.Chen@HorizonNB.Ca
Assistant Professor, Department of Pathology, Dalhousie University
